# *In Situ* Identification of Paleoarchean Biosignatures Using Colocated Perseverance Rover Analyses: Perspectives for *In Situ* Mars Science and Sample Return

**DOI:** 10.1089/ast.2022.0018

**Published:** 2022-09-05

**Authors:** Keyron Hickman-Lewis, Kelsey R. Moore, Joseph J. Razzell Hollis, Michael L. Tuite, Luther W. Beegle, Rohit Bhartia, John P. Grotzinger, Adrian J. Brown, Svetlana Shkolyar, Barbara Cavalazzi, Caroline L. Smith

**Affiliations:** ^1^ Department of Earth Sciences, The Natural History Museum, London, United Kingdom.; ^2^ Dipartimento di Scienze Biologiche, Geologiche e Ambientali, Università di Bologna, Bologna, Italy.; ^3^ NASA Jet Propulsion Laboratory, Pasadena, California, USA.; ^4^ Division of Geological and Planetary Sciences, California Institute of Technology, Pasadena, California, USA.; ^5^ Photon Systems Inc., Covina, California, USA.; ^6^ Plancius Research, Severna Park, Maryland, USA.; ^7^ Department of Astronomy, University of Maryland, College Park, Maryland, USA.; ^8^ Planetary Geology, Geophysics and Geochemistry Lab, NASA Goddard Space Flight Center, Greenbelt, Maryland, USA.; ^9^ Department of Geology, University of Johannesburg, Johannesburg, South Africa.; ^10^ School of Geographical and Earth Sciences, University of Glasgow, Glasgow, United Kingdom.

**Keywords:** Mars, Astrobiology, Mars 2020, Biosignatures, Buck Reef Chert, Microbial mats

## Abstract

The NASA Mars 2020 Perseverance rover is currently exploring Jezero crater, a Noachian–Hesperian locality that once hosted a delta–lake system with high habitability and biosignature preservation potential. Perseverance conducts detailed appraisals of rock targets using a synergistic payload capable of geological characterization from kilometer to micron scales. The highest-resolution textural and chemical information will be provided by correlated WATSON (imaging), SHERLOC (deep-UV Raman and fluorescence spectroscopy), and PIXL (X-ray lithochemistry) analyses, enabling the distributions of organic and mineral phases within rock targets to be comprehensively established. Herein, we analyze Paleoarchean microbial mats from the ∼3.42 Ga Buck Reef Chert (Barberton greenstone belt, South Africa)—considered astrobiological analogues for a putative ancient martian biosphere—following a WATSON–SHERLOC–PIXL protocol identical to that conducted by Perseverance on Mars during all sampling activities. Correlating deep-UV Raman and fluorescence spectroscopic mapping with X-ray elemental mapping, we show that the Perseverance payload has the capability to detect thermally and texturally mature organic materials of biogenic origin and can highlight organic–mineral interrelationships and elemental colocation at fine spatial scales. We also show that the Perseverance protocol obtains very similar results to high-performance laboratory imaging, Raman spectroscopy, and μXRF instruments. This is encouraging for the prospect of detecting microscale organic-bearing textural biosignatures on Mars using the correlative micro-analytical approach enabled by WATSON, SHERLOC, and PIXL; indeed, laminated, organic-bearing samples such as those studied herein are considered plausible analogues of biosignatures from a potential Noachian–Hesperian biosphere. Were similar materials discovered at Jezero crater, they would offer opportunities to reconstruct aspects of the early martian carbon cycle and search for potential fossilized traces of life in ancient paleoenvironments. Such samples should be prioritized for caching and eventual return to Earth.

## 1. Introduction

Identifying and characterizing traces of life throughout the Solar System are among the fundamental challenges facing astrobiology. The search for life beyond Earth is focused on Mars, which is uniquely compelling in that it appears to have been habitable during its early history (the Noachian–Hesperian period; 4.1–3.2 Ga), when it was warmer and stable bodies of water existed at its surface (Carr and Head, 2010; Westall *et al.,* 2013; Cockell, 2014; Grotzinger *et al.,* 2014, 2015). In this regard, the NASA Mars 2020 Perseverance rover mission has as its principal objectives the reconstruction of Noachian–Hesperian paleoenvironments at an ancient martian locality, Jezero crater (Isidis Planitia), and the potential identification of fossil traces of life (biosignatures) in sedimentary strata therein (Williford *et al.,* 2018; Farley *et al.,* 2020). Through sampling and caching a diverse suite of geological samples within hermetically sealed tubes, Mars 2020 also represents the first step toward near-future international collaborations on Mars Sample Return (MSR) (Farley *et al.,* 2020; Grady, 2020; Meyer *et al.,* 2022). Potential organic- and biosignature-bearing sedimentary rocks are high-priority targets for MSR since they may answer major outstanding questions in astrobiology, including (i) the nature of habitable niches early in Mars' history; (ii) whether biosignatures and/or (pre)biotic organic materials are preserved on Mars; and (iii) if life emerged on Mars, for how long the biosphere persisted (Grotzinger *et al.,* 2014; Herd *et al.,* 2021). Such discoveries would address wider questions in astrobiology concerning the nature and distribution of life in the Solar System and would help constrain the early planetary conditions, evolution, and the habitability of rocky bodies (see reviews in Lammer *et al.,* 2009; Cockell *et al.,* 2016).

The Perseverance rover is equipped with a synergistic suite of highly complementary instruments capable of characterizing the geology of Jezero crater at scales from kilometer to micron. Geomorphology and outcrop-scale features are observed collaboratively by the cameras of MastCam-Z and the SuperCam Remote Micro-Imager (RMI) (Bell *et al.,* 2021; Wiens *et al.,* 2021), and subsurface geology is defined by the RIMFAX ground-penetrating radar (Hamran *et al.,* 2020). Mineralogy is determined remotely by MastCam-Z multispectral imaging, SuperCam LIBS, VISIR, and Raman spot analyses (Bell *et al.,* 2021; Wiens *et al.,* 2021). Proximal science is then conducted using the rover arm, on which are mounted SHERLOC (Scanning Habitable Environments with Raman and Luminescence for Organics and Chemicals), a deep-UV Raman and fluorescence spectrometer, and PIXL (Planetary Instrument for X-ray Lithochemistry), an X-ray fluorescence spectrometer (Allwood *et al.,* 2020; Bhartia *et al.,* 2021). SHERLOC has two high-resolution imagers: the Wide-Angle Topographic Sensor for Operations and eNgineering (WATSON), which is similar to the MAHLI multi-scale color camera on the Mars Science Laboratory rover, and the Autofocus and Contextual Imager (ACI), a grayscale fixed focus high-resolution imager for the colocation of laser-induced spectra to rock textures. Through imaging, SHERLOC and PIXL are capable of conducting colocated deep-ultraviolet (DUV) Raman and fluorescence mapping and X-ray elemental lithochemistry analyses, thereby enabling micron-scale correlations between rock fabric and texture; organic content; elemental, chemical, and mineral phase distributions. Once a high-priority sample is identified through use of Mastcam-Z and SuperCam analysis, the science team makes the decision whether to perform proximity science. The rover is placed into position to analyze a rock target with the proximity instruments and potentially collect a core for eventual sample return. Prior to SHERLOC and PIXL analyses, the abrasion tool and gas Dust Removal Tool (gDRT) create a fresh surface of 45 mm in diameter and up to 13 mm deep. The abrading bit produces a smooth surface with a relief of <250 microns to enable in-focus maps and co-registration of SHERLOC and PIXL. Proximal science operations are also governed by limitations in the rover slip potential, tip-tilt, and the geomorphology of the target.

For astrobiological research, the analytical process conducted by Perseverance seeks to identify rocks with high biosignature preservation potential, both for morphological and chemical relics of ancient life. Biosignature preservation potential varies markedly as a function of the formational and diagenetic histories of the host rock (Westall *et al.,* 2015, 2021; Hays *et al.,* 2017); however, it is widely accepted that fine-grained or finely crystalline rocks undergoing early and rapid mineralization or cementation create taphonomic windows of preservation for organo-sedimentary structures, organic materials, (extra)cellular remains, and in exceptional circumstances cellular body fossils (*e.g.,* Anderson *et al.,* 2011; Wacey *et al.,* 2014; Westall *et al.,* 2015; Hickman-Lewis *et al.,* 2018a, 2020; McMahon *et al.,* 2018). Indeed, such lithofacies in Gale crater feature the preservation of organic materials (Freissinet *et al.,* 2015; Eigenbrode *et al.,* 2018). For this reason, an array of likely fine-grained sediments in Jezero crater, mostly associated with the western delta and its remnant buttes, are considered essential MSR targets and will form a crucial part of the sample cache gathered during the Perseverance rover mission (Williford *et al.,* 2018; Herd *et al.,* 2021; Mangold *et al.,* 2021).

The biogeochemistry of a potential fossil martian biosphere remains a topic of scientific hypothesis; however, some general expectations include that it would inhabit aqueous environments, bear similarity to relatively “primitive” bacteria and archaea (photoautotrophs, chemoautotrophs, and/or chemolithotrophs), and exhibit polyextremophily and/or polextremotolerance (Schulze-Makuch *et al.,* 2008; Westall *et al.,* 2015; Cavalazzi *et al.,* 2019). In this regard, the earliest traces of life on Earth, dating to almost 3.5 Ga (*e.g.,* Javaux, 2019 and references therein) are considered excellent potential analogues for the types of ecosystems that may once have colonized habitable environments on Noachian–Hesperian Mars. Controversies surrounding the most ancient traces of life (Brasier *et al.,* 2006; Brasier and Wacey, 2012) mean that such fossils also serve as a benchmark for the stringency and rigor of analysis required to establish the origins of putative martian biosignatures.

**FIG. 1.**
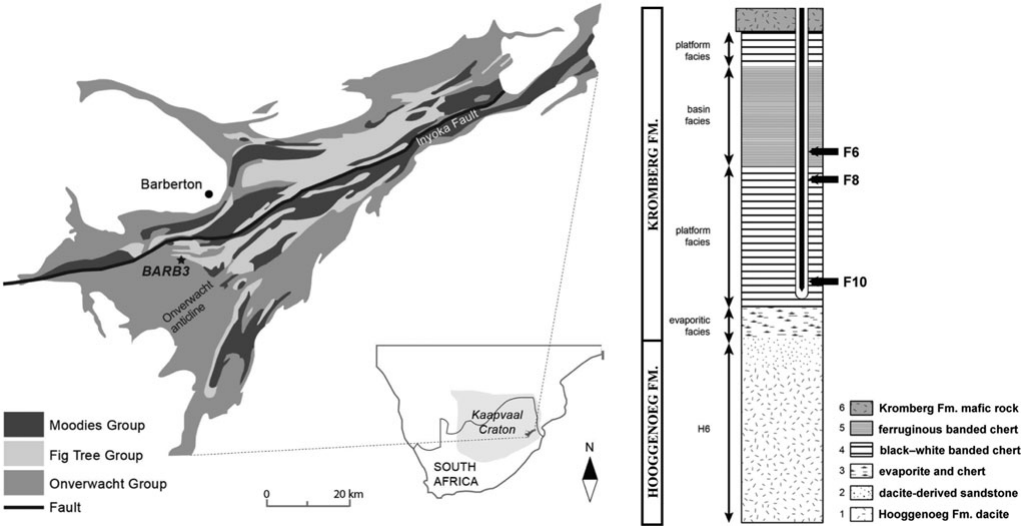
Geological and stratigraphic context of the studied Buck Reef Chert samples. Simplified geological map of the Barberton greenstone belt showing the location of the BARB-3 core, from which the studied samples were sourced; inset shows location within southern Africa. Simplified stratigraphic log of the BARB-3 core showing the position of the studied samples within the basinal (F6) and lower platformal (F8, F10) facies. Modified from Greco *et al.* (2018).

**FIG. 2.**
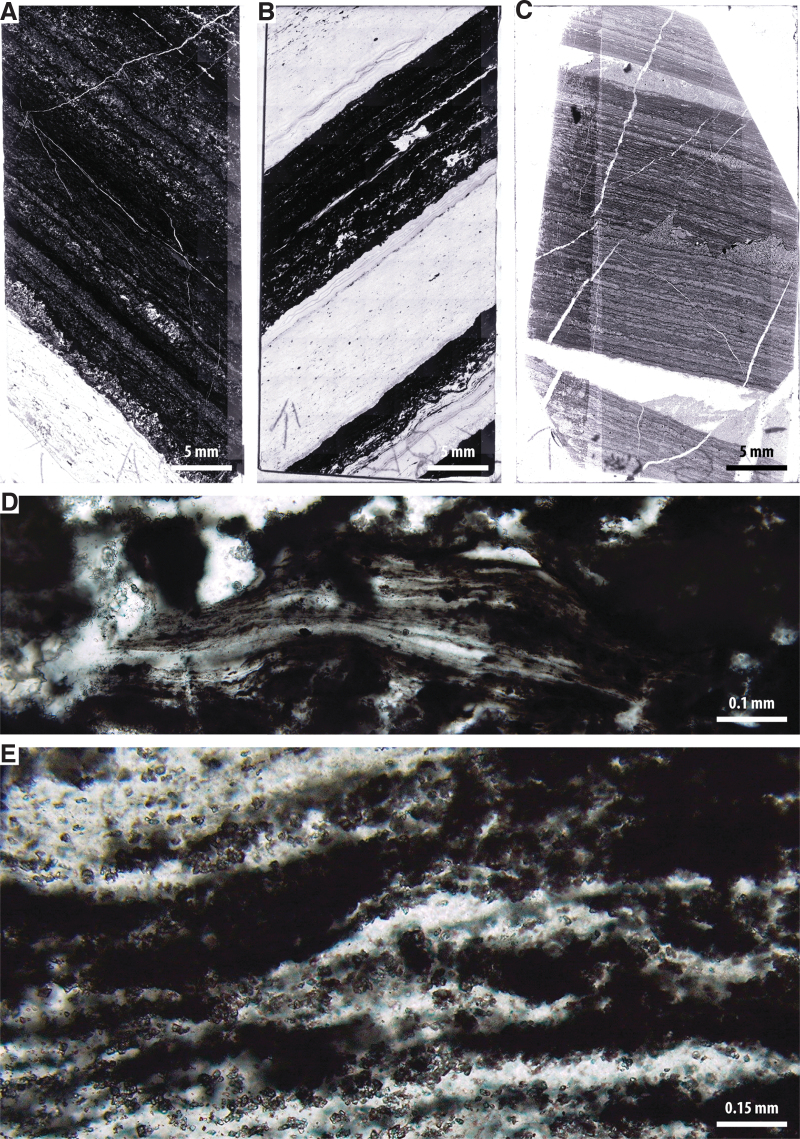
Buck Reef Chert microbial mat fabrics. (**A**) Overview image of thin section F10. (**B**) Overview image of thin section F8. (**C**) Overview image of thin section F6. (**D**) Carbon-rich microbial mats in sample F10. (**E**) Siderite-replaced microbial mats in sample F6.

Correlative microanalysis, that is, the systematic acquisition of spatially colocated, mutually corroborative data, has emerged as a compelling approach in the evaluation of the biogenicity, metabolic networks, and taphonomy of microbial biosignatures in deep time (Brasier *et al.,* 2015; Hickman-Lewis *et al.,* 2019; Wacey *et al.,* 2019; Cavalazzi *et al.,* 2021) and is expected to be an essential strategy in the search for life on Mars. WATSON, SHERLOC, and PIXL can correlatively study mineralogical, molecular, and elemental distributions and concentrations in rock targets on Mars; these detections can be ascribed to specific phases and textures, and this can be considered a planetary science equivalent of terrestrial correlative microanalysis. Herein, we analyze three samples of microbial mat–rich chert from the 3.42 Ga Buck Reef Chert (Barberton greenstone belt, South Africa; Fig. 1) using WATSON, SHERLOC, and PIXL emulator instruments and a Perseverance-representative analytical strategy. Since the Buck Reef Chert contains uncontroversial evidence of a Paleoarchean microbial ecosystem and is characterized by exceptional preservation of fine-scale organic fabrics (Fig. 2) interpreted as relics of productive phototrophic microbialites (Walsh, 1992; Tice and Lowe, 2004, 2006; Tice, 2009; Greco *et al.,* 2018), the microstructures within are excellent astrobiological analogues for the primitive microbial consortia possible within the constraints of Noachian habitability and biogeochemistry. This study aims (i) to demonstrate the utility of correlated SHERLOC and PIXL analyses on astrobiologically relevant materials for Mars by obtaining a dataset similar to that acquired on Mars in advance of sampling; (ii) to address the challenge of biosignature detection and interpretation using *in situ* approaches on Mars; and (iii) to briefly highlight some of the similarities and differences between *in situ* and laboratory datasets with reference to the Perseverance rover mission and present, non-exhaustively, how complementary analyses might be conducted following sample return. Our findings thus have direct bearing on the major science goals of both the mission and sample return: to detect organic, chemical, and mineralogical biosignatures (distribution, composition, and texture) in their local paleoenvironmental context.

## 2. Materials and Methods

### 2.1. Geological context and microbial paleontology

The studied samples (F10, F8, and F6) were obtained from the BARB-3 core collected during the International Continental Scientific Drilling Program (ICDP) Barberton Drilling Project “Peering into the Cradle of Life” (Fig. 1), the stratigraphy and microbial mat fabrics of which were presented in Hofmann *et al.* (2013) and Greco *et al.* (2018). The Buck Reef Chert is the lowermost unit of the Kromberg Formation (Onverwacht Group, Barberton greenstone belt) and comprises four lithofacies: (i) a basal evaporite facies; (ii) the lower platformal facies; (iii) the basinal facies; and (iv) the upper platform facies, overlain by volcanic strata of the Kromberg Formation (Tice and Lowe, 2004, 2006; Hofmann *et al.,* 2013).

Detailed descriptions of the morphologies and preservation of Buck Reef Chert microbial mats show that these mats comprise carbonaceous laminae that, at thin section-scale, form thick sequences of crinkly–wavy carbonaceous laminations intercalated with layers of fine volcanogenic sediment and microcrystalline chemical chert (Tice and Lowe 2006a; Greco *et al.,* 2018). Individual laminations comprise stacks of finely layered carbonaceous materials consistent with biofilm growth (Fig. 2). Siderite and pyrite are intimately associated with the microbial mats, most likely due to heightened ambient concentrations of ferrous iron at the time of deposition (Beukes, 2004; Tice and Lowe, 2004). Trace and rare earth element studies through black and white banded cherts in the Buck Reef Chert imply that the paleoenvironment was either a near-continental platform (Tice and Lowe, 2004) or an epeiric sea (Grosch *et al.,* 2011) nourished by inputs from hydrothermal effluent that presumably contributed to the development of significant biomass (Hofmann and Bolhar, 2007; Ledevin *et al.,* 2019). Bulk carbon isotope values from mat-rich chert range from -36.9‰ to -20.1‰ (mean = -29.9‰), implying biological processing with major contributions from photosynthetic microbes employing hydrogen-based carbon fixation (Tice and Lowe, 2006b), though likely with contributions from secondary heterotrophic reworking of biomass. The mats also exhibit plastic deformation indicative of a primary EPS-bound composition, as well as erosional phenomena, such as roll-ups and tear-ups, consistent with the reworking of microbial mats under wave and current activity (Simonson and Carney, 1999; Tice and Lowe, 2004). Combined, this evidence is consistent with dominantly photosynthetic mat growth under variable energy regimes that encouraged both growth and degradation of mats (*cf*. Tice and Lowe, 2004; Tice, 2009; Hickman-Lewis *et al.,* 2016, 2018b).

**FIG. 3.**
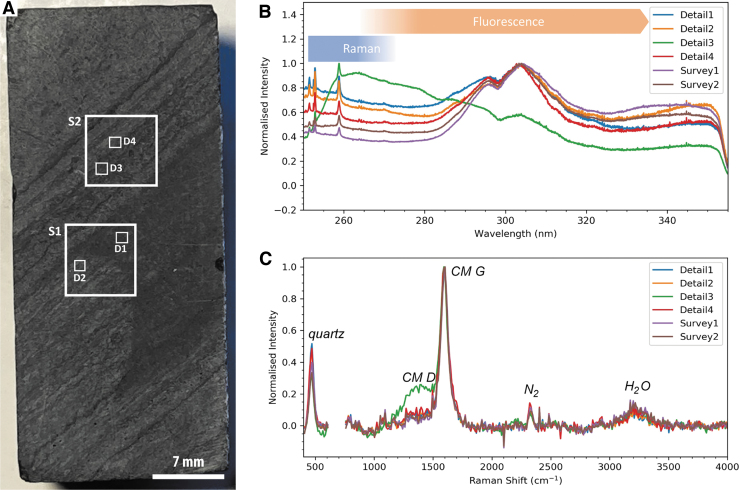
Overview of SHERLOC analyses conducted on sample F10. (**A**) Simulated WATSON image of the sample showing the locations of SHERLOC survey scans (S1–2) and detail scans (D1–4). (**B**) Full SHERLOC spectra (Raman plus DUV fluorescence) for each region of interest. (**C**) Raman spectra for each region of interest.

The thin sections shown in Fig. 2A–2C correspond to the core samples shown in subsequent figures. Optical microscopy observations of these thin sections confirm that they contain well-preserved microbial mats that are either kerogen-rich (Fig. 2D) or siderite-replaced (Fig. 2E). Of the studied samples, F10 and F8 are dominated by kerogen-rich microbial mats, whereas F6 contains a greater amount of siderite in association with mats. Samples for SHERLOC- and PIXL-like analyses were prepared as roughly cut core fragments (*e.g.,* Fig. 3A) as a broad approximation of the abrasion process conducted by the Perseverance rover. Samples were not polished after cutting, and residual damage to the analyzed surface was left unmitigated; this is analogous to the imperfect nature of abrasion patches made by Perseverance, where chatter marks and other abrasion-related surface phenomena mildly disrupt rock fabrics and textures. Prior to analysis, samples were cleaned with isopropyl alcohol and rinsed with ultrapure water.

### 2.2. SHERLOC-like deep-UV Raman and fluorescence spectroscopy

SHERLOC-like measurements were conducted using the SHERLOC Brassboard instrument at the NASA Jet Propulsion Laboratory. The Brassboard is an optical analogue of the SHERLOC flight instrument, adapted to work under terrestrial conditions. Like the flight instrument, the Brassboard generates spatially resolved spectral maps of laser-induced fluorescence and Raman scattering, along with co-boresighted high-resolution (10.1 μm/pixel) microscopy images. The Brassboard uses a pulsed NeCu 248.6 nm laser as the excitation source, firing 40 μs pulses at 80 Hz, focused to a nominal working distance of 48 mm using a SHERLOC-equivalent *f*/7 objective lens. The focused laser spot is annular in shape, with an outer diameter of 110 μm and an estimated illuminated area of 7540 μm^2^, delivering an energy dose of 30–45 J/m^2^ per pulse (Bhartia *et al.,* 2021; Razzell Hollis *et al.* 2021). Fluorescence and Raman scattered photons from organics and minerals in the illuminated volume are collected by the same objective lens, dispersed by a diffractive grating, and detected by a multi-stage thermoelectrically cooled (-54°C) commercial Synapse Symphony II (Horiba) housing a 42-10 BIUV 512 × 2048 pixel CCD from e2v (CCD identical to SHERLOC). Similar to the SHERLOC flight spectrometer, the Raman and fluorescence regions are separated on the detector through curved projection of the spectrum onto the CCD for overall instrument compactness and enabled better separation of the weaker Raman scattered photons from the stronger fluorescence photons (Razzell Hollis *et al.,* 2021). The Raman measurements span 246.8–317.8 nm (-290 to 8760 cm^−1^), and fluorescence measurements span 296.2–357.1 nm (6470 to 12200 cm^−1^). The spectral calibration has a mean absolute error of 0.018 ± 0.010 nm (2.7 ± 1.6 cm^−1^) in the Raman region, assessed using spectral standards of acetonitrile, powdered calcite, highly oriented pyrolytic graphite, and a secondary laser emission line at 252.93 nm (Razzell Hollis *et al.,* 2021).

Each sample was characterized following a similar methodology to SHERLOC operations *in situ* on Mars. First, a survey scan (36 × 36 points, 7 × 7 mm, 100 pulses per point, 200 μm spacing between points) was performed to rapidly map strong Raman and fluorescence signals across a large area. Then, one or more regions of interest were selected based on visible morphology and fluorescence, and a detail scan (10 × 10 points, 1 × 1 mm, 800 pulses per point, 100 μm spacing between points) was performed in each region to maximize signal from lower-yield Raman scattering and obtain a representative Raman spectrum at higher spatial resolution. High-resolution laboratory maps of 4800 points across a larger area (16 × 3 mm) with 100 μm spacing, at 100 pulses per point, were also acquired.

Data processing was conducted by using a combination of custom software and python scripts, utilizing the SciPy, NumPy, and LMFIT packages (Jones *et al.,* 2001; Van der Walt *et al.,* 2011; Newville *et al.,* 2014). Cosmic rays were removed automatically using a pre-established algorithm (Uckert *et al.,* 2019); then Raman and fluorescence spectra were recombined after trimming regions containing only dark noise. For Raman peak analysis, the background signal was removed by subtraction of a polynomial baseline between 0 and 4000 cm^−1^; then any Raman peaks more than >3 times the background noise were fitted using Gaussian functions to determine their precise positions and intensities. The 600–800 cm^−1^ region was ignored due to overlap with the secondary laser emission line at 252.93 nm (692 cm^−1^). Spectral intensity maps were generated by taking the summed intensities of three spectral bands in each spectrum and assigning those intensities to the R, G, B values of the corresponding pixel, normalized to the 2% and 98% percentiles for all three bands across the entire map. For fluorescence maps, R, G, B values represent the 335–345 nm, 305–315 nm, and 275–285 nm bands, respectively; for Raman maps, R, G, B values represent the 1500–1700 cm^−1^, 900–1100 cm^−1^, and 425–475 cm^−1^ bands. Quartz/organic ratio maps were generated by dividing the quartz signal at 450 cm^−1^ by the summed signal of quartz at 450 cm^−1^ and organic material at 1600 cm^−1^. A dip in fluorescence signal was observed at 297 nm in all spectra acquired on the Brassboard and attributed to an instrumental artifact.

### 2.3. PIXL-like elemental mapping

PIXL-like analyses were conducted on a Bruker M4 Tornado μX-Ray Fluorescence spectrometer instrument at the California Institute of Technology Geology and Planetary Sciences Department. All analyses were conducted at 2 mbar pressure, both because conducting X-ray fluorescence (XRF) under low pressure to vacuum conditions allows for better detection of lighter elements and because this closely approximates PIXL-style analyses conducted on Mars where surface pressures are ∼6 mbar. All analyses were conducted using two silicon drift detectors with X-ray tube voltages of 50 kV and currents of 400 μA with no primary beam filters. Detectors were set to measure 90 keV and 130 kcps. Large area maps were analyzed using high-resolution mapping with a 20 μm spot size. Regions of interest (colocated with SHERLOC-like microspectroscopy) and line scans were analyzed using a 120 μm spot size to closely replicate PIXL analyses conducted *in situ* on Mars. Data were analyzed using Bruker M4 Tornado software.

## 3. Results

### 3.1. *In situ* Perseverance-like protocol

In this study, we aimed to closely replicate the analysis of abrasion patches by the Perseverance rover. It is important to note that each abrasion patch on Mars is analyzed in a slightly different manner due to the inherent limitations of rover operations (time of day chosen for light conditions and detector temperature, state of rover charge, availability of high-priority targets, and data volume restrictions), and thus the concept of routine analysis used herein should be taken as notional. Nonetheless, the strategy of analysis applied herein bears crucial similarities to Perseverance operations as follows: (i) instrumental acquisition parameters were kept identical or highly similar to those of SHERLOC and PIXL operating on Mars; (ii) a small number of regions of interest were studied, reflecting the limited time and power available for such analytical procedures on Mars; (iii) SHERLOC conducts nested analyses, starting with large-area survey scans before performing detail analyses on points of interest; and (iv) SHERLOC and PIXL can conduct colocated analyses using the same analytical footprint. As such, the datasets reported herein can be considered representative of those we would expect from similar geological materials on Mars.

#### 3.1.1. Sample F10

##### WATSON-like imaging

**FIG. 4.**
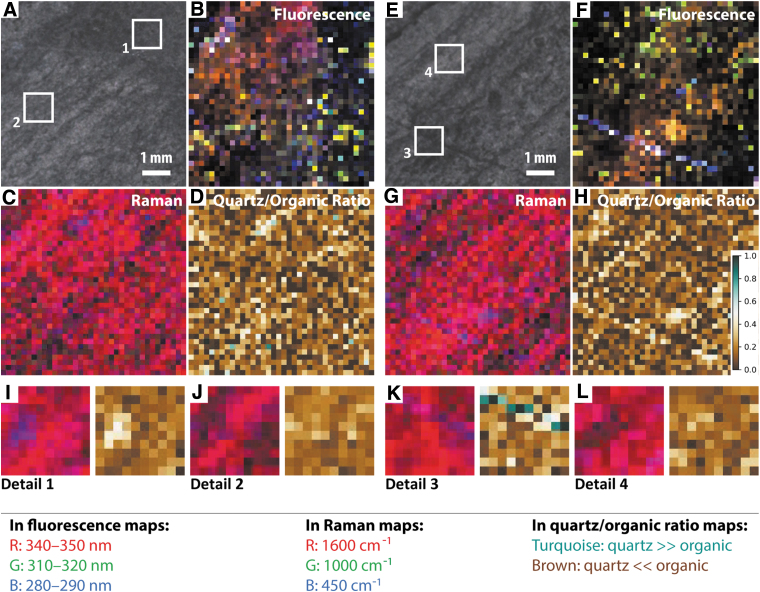
Simulated SHERLOC analyses of sample F10. (**A**–**D**) WATSON-like image and fluorescence, Raman, and Raman quartz/organic ratio maps for survey scan S1. (**E**–**H**) WATSON-like image and fluorescence, Raman, and Raman quartz/organic ratio maps for survey scan S2. (**I**–**J**) Raman and Raman quartz/organic ratio maps for SHERLOC details 1 and 2; see (A) for localization. (**K**–**L**). Raman and Raman quartz/organic ratio maps for SHERLOC details 3 and 4; see (E) for localization. See legend for color code used in this and all subsequent DUV Raman and fluorescence maps. Scale shown in (H) is applicable to all quartz/organic ratio maps.

Images obtained of the core sample and regions of interest (*e.g.,* Figs. 3A, 4A, 4E) show a regularly laminated structure comprising thin dark-toned layers intercalated with thin and thick light-toned layers. The dark-toned layers are characterized by a relatively homogeneous appearance, whereas the light-toned layers feature some apparent granularity. Some folding and undulatory character is exhibited by laminations in the lower portion of the sample (Fig. 3A).

##### SHERLOC-like deep-UV Raman and fluorescence spectroscopy

Two SHERLOC survey scans (7 × 7 mm) were acquired to characterize millimeter-scale organic variability, within which four SHERLOC detail scans (1 × 1 mm) were obtained to reveal fine-scale heterogeneity (Figs. 3 and 4). DUV fluorescence is generally dominated by a peak centered on ∼305 nm, except detail 3, which is dominated by a peak at ∼270 nm (Fig. 3B). Fluorescence mapping highlights three signatures with maxima at ∼300, ∼340, and ∼270 nm; the 340 nm signal shows correlation with laminations (Fig. 4). Detail 2 may show evidence for sp^2^-bonded carbon spectrum in the form of a UV edge of a feature tail that peaks beyond 360 nm (Fig. 3B).

The corresponding Raman spectra show peaks for quartz (450 cm^−1^) and carbonaceous materials (the carbon G-band at ∼1600 cm^−1^) (Fig. 3C). A weak peak at ∼1080 cm^−1^ is interpreted as carbonate; this wavenumber is low relative to the dominant Raman signal of calcite (∼1089 cm^−1^) and dolomite (∼1097–1099 cm^−1^) and most likely arises from siderite. We acknowledge an additional complication in the interpretation of Fe carbonates: the overlap between a carbonate peak at 1080 cm^−1^ and minor peaks of quartz at 1084 and 1165 cm^−1^. Without detection of other carbonate modes, this peak might be considered most consistent with quartz, but when combined with detection of Fe by the PIXL analogue instrument (and its insensitivity to C, see below), Fe carbonate is the most plausible explanation. The spectra within detail 3 also exhibit the carbon D-band at ∼1350 cm^−1^. Raman spectroscopic mapping highlights the dominance of the G-band signature throughout the sample (Fig. 4), which shows a broad correlation with the trend and spatial distribution of dark-toned layers, that is, their intercalation with thin quartz laminae.

Survey 2 and detail 3 show that the 270 nm DUV fluorescence and 1350 cm^−1^ Raman signals are strongly colocated with a cross-cutting vein, which is also enriched in quartz (at 450 cm^−1^) relative to the surrounding matrix (Figs. 3, 4E–4H).

##### PIXL-like elemental mapping

**FIG. 5.**
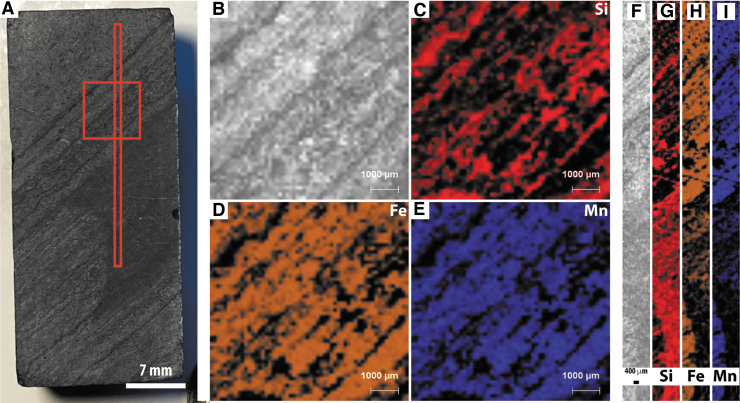
Simulated PIXL analyses of sample F10. (**A**) WATSON-like image showing the location of PIXL scans. (**B**–**E**) PIXL-like scan showing the distributions of Si, Fe, and Mn. (**F**–**I**) PIXL-like line scan showing the distributions of Si, Fe, and Mn.

Two PIXL scans were acquired, one 7 × 7 mm full scan overlapping with SHERLOC survey scan 2 and a line scan through multiple layers of the sample (Fig. 5). Si concentrations are inversely correlated with Fe and Mn, while Fe and Mn are strongly correlated throughout the sample. Ca was detected in trace amounts in the Fe-Mn layers, while S, Al, and Ti were detected in trace amounts but with no clear distributions.

#### 3.1.2. Sample F8

##### WATSON-like imaging

Similar to F10, sample F8 exhibits regions of intercalated undulatory dark-toned and light-toned laminations; however, thick light-toned laminations between the darker regions of this sample exhibit a more homogeneous texture consistent with a finer degree of granularity.

##### SHERLOC-like deep-UV Raman and fluorescence spectroscopy

**FIG. 6.**
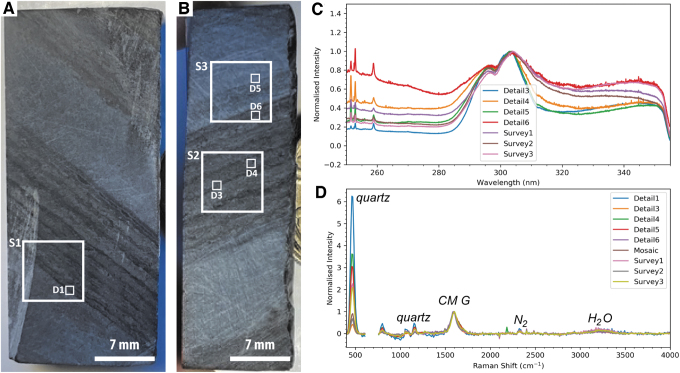
Overview of SHERLOC analyses for sample F8. (**A**–**B**) Simulated WATSON images of two faces of the sample showing the locations of SHERLOC survey scans (S1–3) and detail scans (D1–6). (**C**) Full SHERLOC spectra of each region of interest. (**D**) Raman spectra of each region of interest. Note the absence of the D-band in all Raman spectra.

**FIG. 7.**
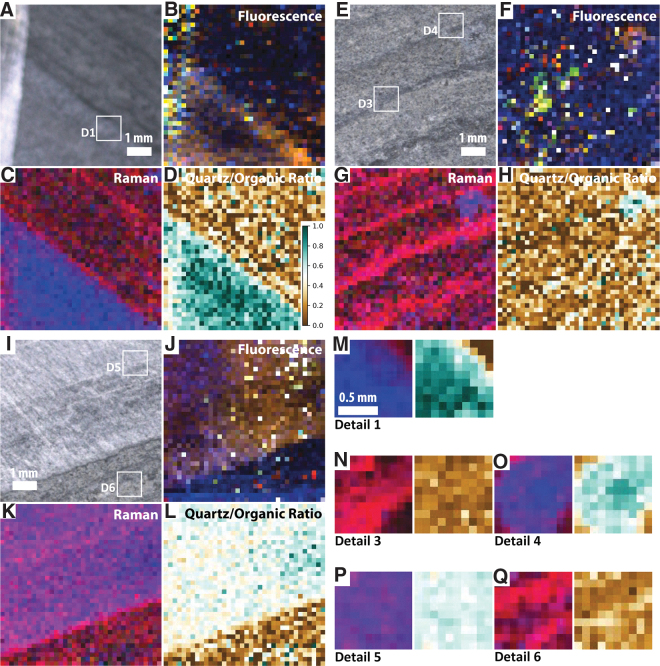
Simulated SHERLOC analyses of sample F8. (**A**–**D**) WATSON-like image and fluorescence, Raman, and Raman quartz/organic ratio maps for survey scan S1. (**E**–**H**) WATSON-like image and fluorescence, Raman, and Raman quartz/organic ratio maps for survey scan S2. (**I**–**L**) WATSON-like image and fluorescence, Raman, and Raman quartz/organic ratio maps for survey scan S3. (**M**) Raman and Raman quartz/organic ratio maps for SHERLOC detail 1; see (A) for localization. (**N**–**O**). Raman and Raman quartz/organic ratio maps for SHERLOC details 3 and 4; see (E) for localization. (**P**–**Q**). Raman and Raman quartz/organic ratio maps for SHERLOC details 5 and 6; see (I) for localization. Scale shown in (D) is applicable to all quartz/organic ratio maps.

Three SHERLOC survey scans and five SHERLOC detail scans were obtained (Figs. 6 and 7), within which fluorescence is centered on a dominant peak at ∼305 nm and a weaker peak at ∼340 nm (Fig. 6B). In survey 1, distinct fluorescence signals are associated with primary laminations and secondary damage, probably due to cutting or abrasion of the surface; the former is better-defined than the latter (Fig. 7A–7D). Survey 2 also shows a moderate correlation between fluorescence signals at 305 nm and two dark-toned laminations (Fig. 7E–7H).

The corresponding Raman measurements show the carbonaceous material G-band, which is enriched in the dark-toned layers, and quartz, which is enriched in the lighter-toned intercalated layers (Figs. 6 and 7). A weak peak at ∼1080 cm^−1^ is again interpreted as carbonate, likely siderite. In survey 1, the Raman signal shows limited correlation with fluorescence; however, one dark lamination adjacent to the underlying light quartz-rich layer is clearly defined in both maps (Fig. 7A–7D). In survey 2, a clearer correlation between the Raman and fluorescence signals is observed; the Raman organic signal is particularly strongly correlated with visible dark-toned laminations (Fig. 7E–7H).

Two detail scans (3 and 4) within survey scan 2 confirm the correlation of the orange-red fluorescence hotspot (which has a maximum at 305 nm but relatively strong signal >320 nm) with a quartz-dominated Raman signal and the yellow-green fluorescence hotspot (which is dominated by the peak at 305 nm) with an organic-rich Raman signal.

##### PIXL-like elemental mapping

**FIG. 8.**
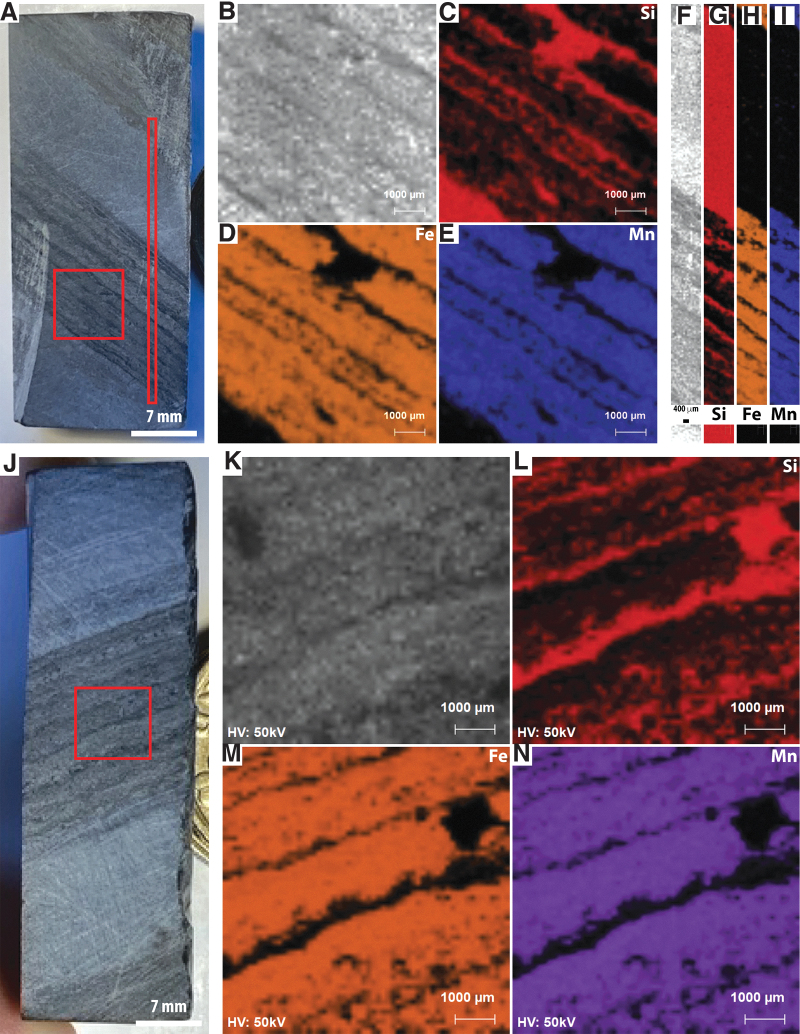
Simulated PIXL analyses of sample F8. (**A**) WATSON-like image showing the location of PIXL scans on face 1. (**B**–**E**) PIXL-like scan showing the distributions of Si, Fe, and Mn. (**F**–**I**) PIXL-like line scan showing the distributions of Si, Fe, and Mn. (**J**) WATSON-like image showing the location of PIXL scans on face 2. (**K**–**N**) PIXL-like scan showing the distributions of Si, Fe, and Mn.

Three PIXL scans were acquired, two 7 × 7 mm full scans overlapping with SHERLOC survey scans 1 and 2 (Fig. 8A–8E, 8J–8N) and a line scan passing through a thick light-toned layer and thick dark-toned layer (Fig. 8F–8I). Si is anti-correlated with Fe and Mn, while Fe and Mn are strongly correlated. Fe-Mn laminae are also found to have small amounts of Ca, while Al and Ti occur in trace quantities as discrete spots throughout the analyzed regions.

#### 3.1.3. Sample F6

##### WATSON-like imaging

In contrast to samples F10 and F8, sample F6 has a faintly laminated texture and is dominated by dark-toned material. One light-toned layer and one gray-toned layer are conformably intercalated with the bulk rock fabric. Several pale cross-cutting veins also obliquely disrupt the primary fabric; however, the sample is otherwise relatively featureless.

##### SHERLOC-like deep-UV Raman and fluorescence spectroscopy

**FIG. 9.**
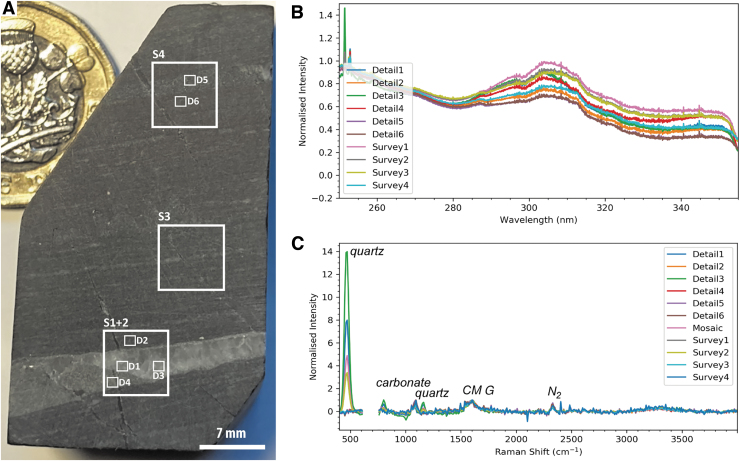
Overview of SHERLOC analyses for sample F6. (**A**) Simulated WATSON image of the sample showing the locations of SHERLOC survey scans (S1–4) and detail scans (D1–6). One pound coin for scale. (**B**) Full SHERLOC spectra of each region of interest. (**C**) Raman spectra of each region of interest. Note the absence of the D-band in all Raman spectra.

**FIG. 10.**
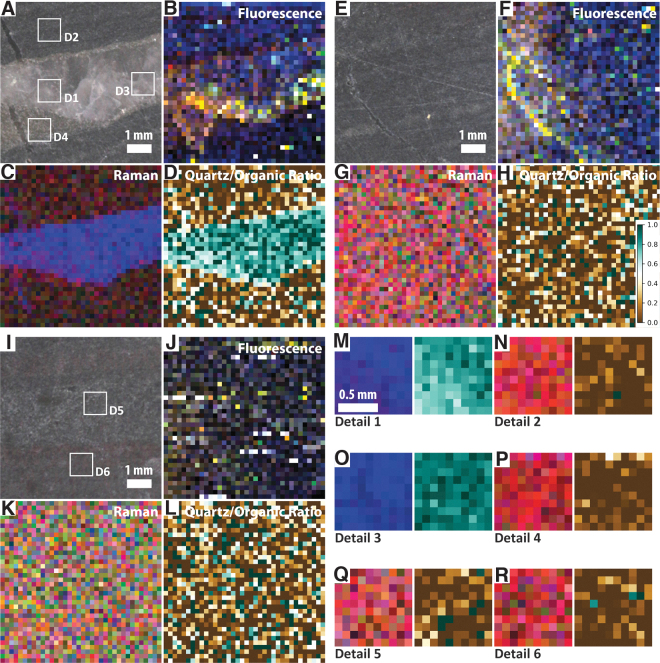
Simulated SHERLOC analyses of sample F6. (**A**–**D**) WATSON-like image and fluorescence, Raman, and Raman quartz/organic ratio maps for survey scan S1. (**E**–**H**) WATSON-like image and fluorescence, Raman, and Raman quartz/organic ratio maps for survey scan S3. (**I**–**L**) WATSON-like image and fluorescence, Raman, and Raman quartz/organic ratio maps for survey scan S4. (**M**–**N**) Raman and Raman quartz/organic ratio maps for SHERLOC details 1 and 2; see (A) for localization. (**O**–**P**). Raman and Raman quartz/organic ratio maps for SHERLOC details 3 and 4; see (E) for localization. (**Q**–**R**). Raman and Raman quartz/organic ratio maps for SHERLOC details 5 and 6; see (I) for localization. Scale shown in (H) is applicable to all quartz/organic ratio maps.

Three survey scans and six detail scans were obtained, all of which exhibit weak fluorescence at ∼310 nm. In all regions, fluorescence is strongest along the fractures, for example, along the edges of the large fracture in survey 1 and throughout the cross-cutting fractures in other regions of interest (Figs. 9 and 10). Dark regions in this sample exhibit relatively limited fluorescence. Detail scans confirm the relative heterogeneity of regions; that is, quartz-rich regions have minimal organic signal, and organic regions have minimal quartz signal.

The corresponding Raman scans show a weak organic signal, quartz, and a more prominent peak at ∼1080 cm^−1^ in regions with weak quartz signals that is again interpreted as carbonate, most likely siderite (Figs. 9 and 10). Unlike samples F10 and F8, the weak organic and quartz signals exhibit no systematic variation within the relatively homogeneous matrix, correlating instead with layering or fractures.

##### PIXL-like elemental mapping

**FIG. 11.**
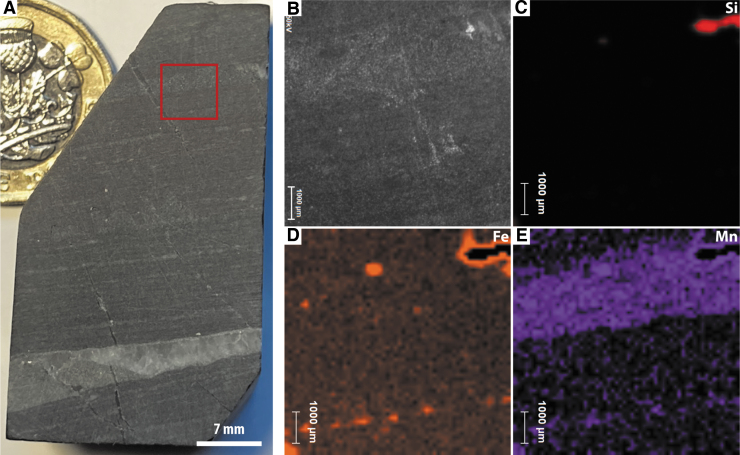
Simulated PIXL analyses of sample F6. (**A**) WATSON-like image showing the location of the PIXL scan. One pound coin for scale. (**B**–**E**) PIXL-like scan showing the distributions of Si, Fe, and Mn.

One 7 × 7 mm PIXL scan was acquired, overlapping with SHERLOC survey scan 4 (Fig. 11). As in samples F10 and F8, Si is anti-correlated with Fe and Mn, while Fe and Mn are strongly correlated, Mn being enriched in certain layers (Fig. 11E).

### 3.2. High-resolution and large-area laboratory characterization

The design and operating conditions of the SHERLOC and PIXL instruments on board the rover introduce limitations in terms of scan area, duration, and spatial resolution. Complementary laboratory analyses using high-resolution Raman spectroscopy and μXRF were undertaken to confirm the results of emulated SHERLOC and PIXL analyses and provide higher-quality data. The higher resolution and improved detection capabilities of laboratory instruments relative to miniaturized rover instruments are more representative of the level of analysis with which core samples may be studied following MSR.

#### 3.2.1. Sample F10

**FIG. 12.**
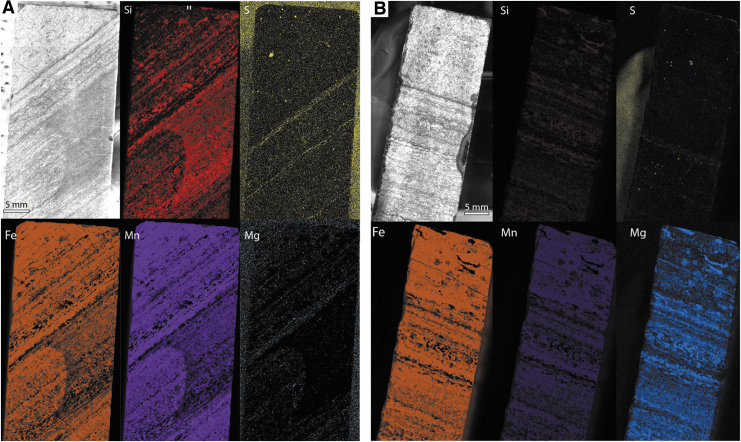
Optical images and laboratory XRF scans for two faces of sample F10. The face analyzed in (**A**) corresponds to Figs. 3–5, while the face in (**B**) was not analyzed using the Perseverance-like protocol.

High-resolution XRF mapping of the whole sample surface at a spatial resolution of 30 μm (Fig. 12) confirmed the spatial correlation of Fe and Mn and the inverse relationship of Si to Fe and Mn. In addition, numerous S-rich laminae and S-rich particles were identified, as well as trace Al, Ca, Ti, and Zn.

#### 3.2.2. Sample F8

**FIG. 13.**
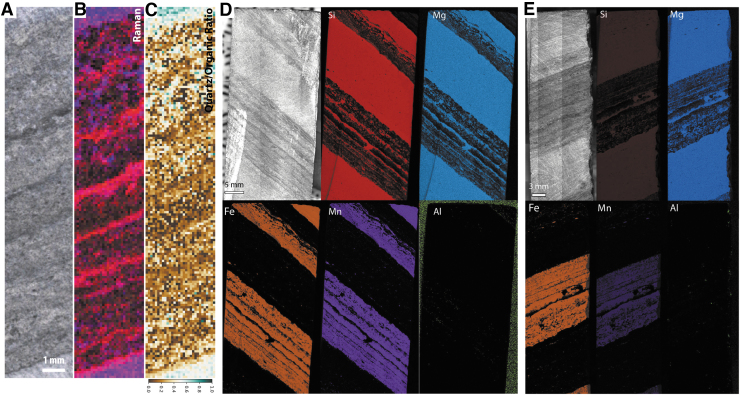
(**A**–**C**) Optical image, laboratory Raman scan, and quartz-organic ratio map for a whole face of sample F8. (**D**–**E**) Optical images and laboratory XRF scans for two faces of sample F8. The face analyzed corresponds to Figs. 6–8.

High-resolution, large-area Raman mapping through a dark-toned region (Fig. 13A–13C) showed a clear arrangement of layers with different intensities of organic signal, the highest intensities localized within the darkest layers, as well as several small quartz-rich domained distributed throughout the region. These maps also show that the light-toned blocks that sandwich the dark region consist mainly of quartz. Mapping the Raman intensity ratio between quartz and organics demonstrates that mid-toned layers have similar relative concentrations of organic material and quartz; this suggests that the Raman signal is being modulated by variation in laser attenuation by the rock rather than concentration. This is consistent with the XRF observation of Fe carbonates in the lower-signal layers, since Fe-rich minerals are known to strongly absorb DUV light (Cloutis *et al.,* 2008; Shkolyar *et al.,* 2018; Carrier *et al.,* 2019).

High-resolution XRF mapping of the whole sample surface (Fig. 13D, 13E) again showed correlation between Fe and Mn, and inverse correlation between Si and Fe/Mn. Some Al-rich laminae were identified, and trace Ca and Ti was noted throughout the sample.

#### 3.2.3. Sample F6

**FIG. 14.**
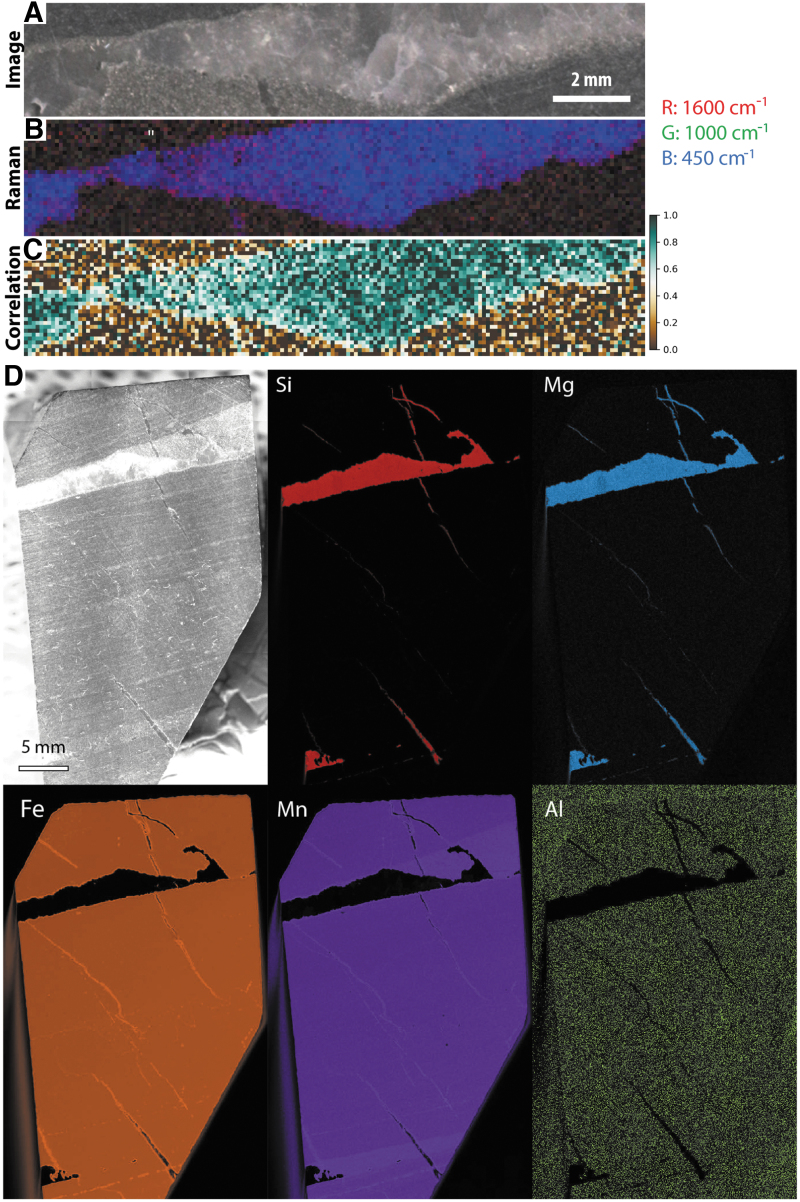
(**A**–**C**) Optical image, laboratory Raman scan, and quartz-organic ratio map for a larger region of interest within sample F6. (**D**) Optical images and laboratory XRF scans for one face of sample F8.

High-resolution, large-area Raman mapping of a contact between light- and dark-toned layers (Fig. 14A–14C) showed that the light-toned layer is generally rich in the quartz signal, while the organic signal is more apparent in the dark-toned region; overall, the signal intensity is lower in the dark-toned region—possibly due to the presence of Fe carbonate (*cf.* XRF maps in Fig. 14D). The more granular mid-toned layer (upper right of Fig. 14A) appears identical to the dark-toned region. A fracture cutting through mid-toned layer is slightly enriched in quartz and was possibly introduced at the same time as the quartz-rich layer.

High-resolution XRF mapping of the whole sample surface showed overlap between very strong signals of Fe and Mn, which anticorrelate with Si. Although Si is present throughout the sample, its relatively high concentrations with the light-toned region (vein) are most visually apparent. Some Al and trace Ca and Ti were also observed throughout the sample.

In summary, throughout the three samples analyzed, strikingly similar signals and identical distributions in organic and elemental lithochemistry were observed, validating the results obtained by the SHERLOC and PIXL emulators.

## 4. Discussion

### 4.1. Mars analogy of the Buck Reef Chert

There is no perfect terrestrial analogue for planetary exploration. Due to variably significant and/or subtle differences in chemistry, mechanical properties, and paleoenvironmental setting, there are always differences between a proposed analogue sample and the extraterrestrial material to which it refers (Foucher *et al.,* 2021). Rather than assuming ideal analogy, samples chosen as analogues should be defined as such on the basis of the characteristics in which they are analogous to the target object and recognized for inevitable shortcomings; such materials are termed “functional analogues” (Foucher *et al.,* 2021).

We consider the Buck Reef Chert to be an appropriate martian astrobiological analogue for the following reasons: (i) Its Paleoarchean age is approximately contemporaneous with the Noachian–Hesperian period during which Mars is considered to have been habitable; thus the biosignatures within can be expected to have attained a similar, but not greater, level of evolutionary sophistication than anticipated biosignatures on Mars (*cf*. Westall *et al.,* 2015). (ii) Its mechanism of preservation by silicification bears similarity to outcrops of proposed astrobiological significance on Mars, among which silica-rich deposits around Jezero crater detected in orbital data (Tarnas *et al.,* 2019) and along the Mars Exploration Rover traverses (Squyres *et al.,* 2008; Ruff and Farmer, 2016) are key examples. (iii) Its carbonate-rich mineralogy and the prevalence of siderite parallel both key outcrops of interest at the Jezero crater margins to be investigated by Perseverance in the near future (Horgan *et al.,* 2020) and the carbonate mineralogies that might be anticipated to occur at the mildly acidic water–rock interfaces of early martian lakes (Agangi *et al.,* 2021). It is further of note that the studied samples were sourced from core samples rather than outcrops and have therefore been spared the vicissitudes of alteration and degradation at Earth's surface and should be expected to retain primary lithochemical characteristics.

There are nonetheless shortcomings to the analogy of the Buck Reef Chert. Firstly, the thermal maturity of the sample (lower greenschist grade metamorphism, as is the case for the entirety of the Barberton greenstone belt) is greater than that expected at the surface of Mars. As such, the biogeochemical signals obtained from our analyses may be considered a “worst-case scenario” for biosignature detection on Mars. Secondly, while silica-rich compositions have been identified in martian rocks (Squyres *et al.,* 2008; Ruff and Farmer, 2016; Tarnas *et al.,* 2019), it is unclear whether these materials are cherts *sensu stricto*. Despite these caveats, for the reasons outlined we consider the BARB-3 core Buck Reef Chert samples to be appropriate astrobiological analogues for Mars.

### 4.2. Biosignature detection

Our emulated WATSON–SHERLOC–PIXL analyses demonstrate that correlation among imaging, organic characterization, and the mapped distribution of organic, mineral, and elemental signatures enables the detection of organic-rich laminated fabrics within a geologically (thermally and diagenetically) mature silica–carbonate matrix. Whether this constitutes a definitive biosignature detection is challenging to evaluate at this stage since the unambiguous identification of a biosignature should rely upon numerous high-resolution datasets, some using techniques incompatible with the power and implementation constraints of rover engineering and technology. Although it is often true that no single analytical dataset can provide a “smoking gun” for the detection of ancient life (*e.g.,* Brasier *et al.,* 2006; Schopf *et al.,* 2010 and references therein), these emulator data acquired under mission-representative conditions show that the analyzed Buck Reef Chert samples bear many of the hallmarks of fossilized microbial mats from shallow-water environments:

- WATSON-like images show that the laminated architecture of the rock comprises regular alternations between dark- and light-toned layers, that is, a microstratigraphy consistent with modern and fossil terrestrial microbialites and biolaminites (*e.g.,* Noffke *et al.,* 2013; Noffke, 2021). Although this microstratigraphy is also consistent with many other finely laminated sediments that show alternating light and dark layers as a result of grain size or mineralogical variations (Grotzinger and Knoll, 1999), the dark-toned laminations exhibit undulatory, non-isopachous morphologies that are largely inconsistent with the isopachous characteristic of abiotic laminations (Grotzinger and Knoll, 1999; Pope *et al.,* 2000; Sugitani *et al.,* 2007; Perri *et al.,* 2013). Nonetheless, thin section preparation following MSR would be required to achieve resolutions enabling the identification of unambiguously biogenic micromorphologies (Fig. 2). The observation of a dominantly fine-grained sedimentary texture also suggests higher biosignature preservation potential (*e.g.,* McMahon *et al.,* 2018; Bosak *et al.,* 2021), although this is again dependent upon mineralogy and rapidity and timing of mineralization.
- SHERLOC-like fluorescence and Raman mapping confirms that the dark-toned layers are carbonaceous in origin and indicates that the organic material is thermally mature (*i.e.,* moderately graphitic carbonaceous materials rich in polyaromatic sp^2^-bonded carbon). As such, the dark-toned layers can be considered syngenetic with the host rock and are of a composition comparable to the Raman-determined kerogenous nature of ancient microbial mats from numerous Precambrian localities (*e.g.,* Schopf *et al.,* 2005; Marshall *et al.,* 2010; Delarue *et al.,* 2016). Cross-cutting fabric relationships between microbial laminations and later veining further enable the sequence of events within the samples to be established; the veins are demonstrably secondary and associated with a distinct silica-enriched lithochemistry and post-depositional, less mature, organic content (*e.g.,* Figs. 3 and 4).
- PIXL-like elemental mapping demonstrates that organic laminations (identified by SHERLOC-like analyses) are predominantly preserved within silica and are intercalated with siderite-rich layers that also contain organic material. These iron carbonate layers are likely a result of growth on a carbonate platform under mildly acidic aqueous chemistry (*cf*. Tice and Lowe, 2004, 2006a; Westall *et al.,* 2018). The ubiquitous presence of laminated microcrystalline silica throughout the samples suggests a rapidly lithifying chemical agent of preservation, again suggesting that silica may have precipitated in the primary environment and acted as a means of early organic preservation.

Our results, following a sequence identical to that of the standard Perseverance characterization protocol prior to sampling events, imply that the detection of possible and probable microbial biosignatures using correlated WATSON–SHERLOC–PIXL data is achievable during *in situ* mission operations. In the event that an unambiguous biosignature identification cannot be made following this protocol, we nonetheless show that materials with high biosignature preservation potential—key targets in the search for astrobiologically relevant samples (Westall *et al.,* 2015; Hays *et al.,* 2017)—can be identified during standard mission operations. Additionally, we highlight that fine-grained, finely laminated, siliceous sedimentary rocks, such as the Buck Reef Chert, may be excellent paleoenvironmental archives and can assist in the reconstruction of the depositional energy, chemistry, and redox of the ambient sub-aqueous environment. If encountered on Mars, similar materials would be paramount candidates for caching and subsequent sample return to Earth.

Laboratory studies carried out to supplement SHERLOC–WATSON–PIXL analyses demonstrate the importance of MSR since these more thorough and higher-resolution analyses will provide pivotal supporting evidence for the characterization and detection of biosignatures upon return to Earth. In the present case, previous laboratory analyses, simulating post-MSR approaches, have already shown that the studied laminated fabrics are of biogenic origin, their exceptional preservation by virtue of early and rapid silicification (Walsh and Lowe, 1999; Tice and Lowe, 2004, 2006a; Greco *et al.,* 2018).

### 4.3. Implementation of the Perseverance protocol

The Perseverance-like characterization presented in this study emulates the final stage of high-resolution analysis during the rover's targeted operation on Mars and is typically conducted on abraded patches. Complementary prior orbital and outcrop studies provide the context to understand the broader paleoenvironment of the sample. Analyses on abraded patches will be supported by this plethora of data from other payload instruments and considerations that facilitate detailed assessments of the origin of a putative biosignature. At this advanced stage of *in situ* characterization, identification of the key attributes noted above would provide a logical motivation for caching in light of the potential presence of a biosignature. Following observations of the Kodiak delta remnant and delta front early in the Mars 2020 prime mission, outcrop-scale observations by Perseverance are already ground-truthing the presence of habitable conditions (a fluvio-deltaic setting) potentially containing fine-grained sediments with probable biosignature preservation potential (Mangold *et al.,* 2021; Williford *et al.,* 2021). Ongoing outcrop characterization throughout the mission will allow the selection of localities with astrobiological potential for analysis and sampling to be refined. Based on the above emulated *in situ* analysis scenario, combined WATSON–SHERLOC–PIXL datasets can be considered a minimum combination of analyses required to make a confident decision for sample caching. Following return to Earth and laboratory analysis, further high-resolution and high-sensitivity analyses on these samples would be conducted to confirm whether or not they represent preserved microbial mats or microbialites/stromatolites, or whether the observed laminated features are abiotic in origin (Cady *et al.,* 2003; Allwood *et al.,* 2015; Hays *et al.,* 2017; Williford *et al.,* 2018; Steele *et al.,* 2022).

**FIG. 15.**
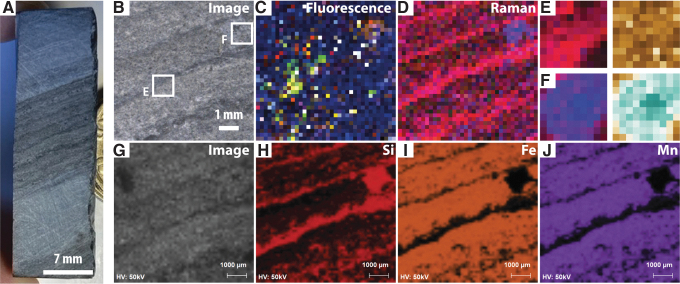
Example simulation of correlated WATSON imaging, SHERLOC DUV-Raman spectroscopy, and PIXL element mapping conducted during a Mars 2020 Perseverance sampling cycle. Analyses were conducted on the same region of interest (sample F8) following an identical protocol to that conducted during each sampling event on Mars. (**A**) WATSON-like image of an abraded sample surface. (**B**–**D**) WATSON-like close-up image with fluorescence, and Raman maps for survey scan 2. (**E–F**) Detail scans 3 and 4 within survey region S2. (**G–J**) PIXL elemental mapping showing the distribution of Si, Fe, and Mn.

Under time-pressured mission conditions and competing science priorities, developing an effective analytical strategy is essential to ensure rapid, yet accurate, assessments of rock fabric and composition; to define specific sampling localities; and to identify potential biosignatures. In this regard, before each sampling activity, Perseverance will execute the Sample Threshold Observation Protocol (STOP) suite of analyses, which is designed to ensure complete fundamental characterization of the sample, thereby ensuring that it meets the optimal criteria prior to a sampling and caching commitment. The presented WATSON, SHERLOC, and PIXL analyses are contained within the STOP list, and Fig. 15 presents an example correlated dataset that could be obtained prior to sampling. Although mission time constraints mean that there will probably not be more than one set of SHERLOC and one set of PIXL analyses in a single STOP list (*e.g.,* the datasets shown in Fig. 15), we strongly advocate for further opportunistic analyses where particularly compelling features are observed, for example, laminated carbonaceous fabrics or sedimentary textures that may have arisen from biological activity.

### 4.4. On Mars as it is on Earth

By comparing the results of a Perseverance-like analytical protocol (Fig. 15) with high-resolution laboratory analyses (Figs. 12–14), it becomes possible to appraise similarities and differences between the characterization possible on Mars versus that possible on Earth, that is, during the mission versus following sample return. Encouragingly, we have shown that, despite the power, resolution, and time constraints imposed by the mission operation protocols simulated, Perseverance-like and laboratory analytical suites reach similar and highly complementary conclusions: the rover-like analyses simulated herein would allow us to reach the conclusion that a sample should be cached because it contains potential biosignatures, while the analysis of those samples on Earth would show that fabrics within these samples are biogenic. The only major differences between the mission-like and laboratory analyses presented are as follows: (i) the impossibility to observe individual microscopic biofilms, for which invasive sample preparation (thin sections) and microscopy are necessary; and (ii) limits of scan size imposed by rover arm instrumental configuration and power constraints. Additionally, the studied samples have undergone a more severe thermal history (lower greenschist grade metamorphism) than is expected at the surface of Mars, and we can therefore anticipate that organic materials on Mars would generally be less degraded than those preserved within the Buck Reef Chert microbialites. Regardless, our study demonstrates that micron- to millimeter-scale correlated analyses on Mars using Perseverance instrumentation can reach a high level of detail in lithological characterization, facilitating the optimal selection of potential biosignature-bearing samples for return to Earth. Such samples would fulfil the major astrobiology goals of the Mars 2020 mission (Williford *et al.,* 2018; Farley *et al.,* 2020) and are certain to provide detailed insights into the geochemistry and elemental cycles, particularly the carbon cycle, of early Mars.

It is probable that samples hosting putative biosignatures will be known long prior to their return to Earth, during which time an appropriate analytical protocol can be developed (*cf*. Rummel and Kminek, 2018; Kminek *et al.,* 2022; Meyer *et al.,* 2022), likely on a sample-by-sample basis. A wide range of returned sample science could be proposed further to *in situ* analyses conducted by Perseverance in order to confirm or disprove their potential biogenic nature. Since the amount of returned sample will be limited, this analytical protocol should focus on obtaining information still missing after Perseverance analyses, that is, to conduct analyses that are impossible when using *in situ* instrumentation. Such data will probably include micromorphological analyses requiring thin section preparation (as shown in Fig. 2) or the extraction of ultrathin rock sections using a focused ion beam (*e.g.*, Cavalazzi, 2007; Schiffbauer and Xiao, 2009; Cavalazzi *et al.,* 2021), isotope geochemistry requiring the identification and isolation of individual mineral phases or fabric elements (*e.g.,* Valley *et al.,* 1994; van den Boorn *et al.,* 2010; Lowe *et al.,* 2020), high-resolution tomography requiring minimally invasive sample preparation (*e.g.,* Hickman-Lewis *et al.,* 2016b, 2019; Maldanis *et al.,* 2020), and organic geochemistry requiring the solvent extraction of organic aliquots (*e.g.,* Duda *et al.,* 2018).

## 5. Conclusions and Perspectives

The Perseverance-like analytical protocol presented here, based on the correlative capability of three instruments aboard the Mars 2020 Perseverance rover, namely WATSON, SHERLOC, and PIXL, can help facilitate the detection of potential biosignatures in a rover-like sampling scenario. We used identical instrumental parameters and targeting strategies as deployed during Perseverance operations. The emulation of WATSON, SHERLOC, and PIXL analyses on Mars allowed an interpretation of carbonaceous cherts of the 3.42 Ga Buck Reef Chert as biosignature-bearing, that is, the studied samples' laminated architecture, composition, and paleoenvironmental signatures are consistent with habitable settings containing mature organic materials of biological origin. This suggests that a similar level of interpretation will be possible at the surface of Mars. Previous, more conventional studies of samples from the Buck Reef Chert confirm that these horizons contain well-preserved organic-rich biosignatures (microbial mats) of Paleoarchean age, which are considered to serve as analogues of possible primitive biosignatures that might be found on Mars. Our protocol captures a high degree of this biogenic interpretation.

Organic compounds formed via microbial metabolism are among the prime targets for sample return, and our study of the Buck Reef Chert suggests that, if such materials exist in the rocks at Jezero crater, they could be identified by using the Perseverance analytical protocol. If similar targets are identified on Mars, they would offer opportunities both to reconstruct aspects of the early martian carbon cycle and to search for potential fossilized traces of life in ancient paleoenvironments. Under such circumstances, the protocol of correlated analyses presented herein could be deployed to prioritize samples for caching and eventual return to Earth.

As the Perseverance rover continues its operations in Jezero crater, numerous studies use datasets based on simulated payload analyses as a means to investigate geological materials encountered and anticipated along its traverse (*e.g.,* Mandon *et al.,* 2021; Razzell Hollis *et al.,* 2021; Anderson *et al.,* 2022) as well as to optimize the treatment and interpretation of datasets under mission conditions and constraints (*e.g.,* Allwood *et al.,* 2020; Flannery *et al.,* 2021). For astrobiology, the obtainment of datasets such as those reported on the Strelley Pool stromatolites (Allwood *et al.,* 2015) and the Buck Reef Chert (this study) will provide guidance for the interpretation of similar biosignatures if encountered on Mars. Such studies also provide initial guidance for sample return. At present, preparations for MSR are being formulated and refined with emphasis on analytical strategies, curation and sample storage, and planetary protection (Rummel and Kminek, 2018; Smith *et al.,* 2021; Kminek *et al.,* 2022; Meyer *et al.,* 2022; Tait *et al.,* 2022). Defining the datasets to which we will have access from only *in situ* rover analyses will facilitate the prioritization of analyses conducted on returned materials and could conceivably incentivize the development of instrumentation or analytical approaches enabling missing data to be acquired. In this regard, the analysis of limited and “precious” early Earth samples is particularly prudent since these materials face many of the same challenges that will be faced by materials returned from Mars: in both cases, it is necessary to extract a maximum amount and quality of data from a minimal volume of sample material, using non-invasive and nondestructive approaches insofar as possible. Further studies using Perseverance-like protocols on similar terrestrial samples should further help constrain the nature and interpretation of data gathered *in situ* on Mars and best prepare astrobiologists for the challenge of assessing potential traces of life in returned martian samples.

## Abbreviations Used

DUV: deep-ultraviolet
MSR: Mars Sample Return
PIXL: Planetary Instrument for X-ray Lithochemistry
SHERLOC: Scanning Habitable Environments with Raman and Luminescence for Organics and Chemicals
STOP: Sample Threshold Observation Protocol
WATSON: Wide-Angle Topographic Sensor for Operations and eNgineering
XRF: X-ray fluorescence
